# Prevalence of insulin resistance and its association with metabolic syndrome criteria among Bolivian children and adolescents with obesity

**DOI:** 10.1186/1471-2431-8-31

**Published:** 2008-08-12

**Authors:** Margoth Caceres, Carlos G Teran, Susana Rodriguez, Marcos Medina

**Affiliations:** 1 Department of General Pediatrics, Centro Pediatrico Albina Patiño, Cochabamba, Bolivia

## Abstract

**Background:**

Obesity is a one of the most common nutritional disorder worldwide, clearly associated with the metabolic syndrome, condition with implications for the development of many chronic diseases.

In the poorest countries of Latin America, malnourishment is still the most prevalent nutritional problem, but obesity is emerging in alarming rates over the last 10 years without a predictable association with metabolic syndrome.

The objective of our study was to determine the association between insulin-resistance and components of the metabolic syndrome in a group of Bolivian obese children and adolescents. The second objective was determining the relation of acanthosis nigricans and insulin-resistance.

**Methods:**

We studied 61 obese children and adolescents aged between 5 and 18 years old. All children underwent an oral glucose tolerance test and fasting blood sample was also obtained to measure insulin, HDL, LDL and triglycerides serum level. The diagnosis of metabolic syndrome was defined according to National Cholesterol Education Program-Adult Treatment Panel (NCEP-ATP III) criteria adapted for children.

**Results:**

Metabolic syndrome was found in 36% of the children, with a higher rate among males (40%) than females (32.2%) (p = 0.599). The prevalence of each of the components was 8.2% in impaired glucose tolerance, 42.6% for high triglyceride level, 55.7% for low levels of high-density lipoprotein cholesterol, and 24.5% for high blood pressure. Insulin resistance (HOMA-IR > 3.5) was found in 39.4% of the children, with a higher rate in males (50%) than females (29%). A strong correlation was found between insulin resistance and high blood pressure (p = 0.0148) and high triglycerides (p = 0.002). No statistical significance was found between the presence of acanthosis nigricans and insulin resistance.

**Conclusion:**

Metabolic syndrome has a prevalence of 36% in children and adolescent population in the study. Insulin resistance was very common among children with obesity with a significant association with high blood pressure and high triglycerides presence.

## Background

Obesity is one of the most common nutritional problems in developed countries. Actually the incidence of obesity is increasing considerably in Latin America countries too.[1] In Bolivian children, malnourishment secondary to poor intake is still the most prevalent nutritional problem, but obesity is emerging in alarming rates between the last 10 years in the most economical productive sectors inside the country. [2,3]

Health care professionals should be concerned about overweight and obesity because of the well established relations between excess body weight and such medical conditions as type 2 diabetes, hypertension, atherosclerotic cardiovascular disease and osteoarthritis.

The metabolic syndrome also called (insulin resistance syndrome and X syndrome) is a common pathophysiologic condition with implications for the development of many chronic diseases. Obesity beginning in childhood often precedes the hyperinsulinemic state. The metabolic syndrome is rapidly increasing in prevalence with rising childhood obesity and sedentary lifestyles worldwide. Although chronic diseases are now well recognized as a growing problem for low- and middle-income countries, limited data are available for these countries, and the developing world has been largely ignored in health strategies. [4]

The insulin resistance is defined as an impaired ability of plasma insulin to promote peripheral glucose disposal, suppress hepatic glucose, and inhibit very low density lipoprotein (VLDL) output. The clinical phenotype of insulin resistance syndrome mainly includes, centrally obesity, acanthosis nigricans (AN), skin tags, striae, acne, hirsutism, hypertension and atherogenic dyslipidemia manifested by increased VLDL, triglycerides and reduced levels of high density lipoprotein (HDL) cholesterol. [5,6]

It is well established that insulin resistance is also present in patients without obesity, whereas obese children can be insulin sensitive. There are no clear genetic studies to support a racial predilection, but actually obesity and insulin resistance syndrome is more frequent in white people and African-Americans people. Africans, Asiatic, Arabic and Latin American race are less commonly affected. Apparently, cultural differences, economical disadvantages and lifestyle behaviors may account for some of the race disparity in obesity-related diseases and disease outcomes, but the rates of obesity are clearly increasing in all groups in the last 20 years. [7-9]

In adults, the definition of insulin resistance syndrome varies in terms of the indicators and cutpoints used. The US National Cholesterol Education Program includes abnormalities in any three of the following factors: glucose level, triglyceride level, high density lipoprotein cholesterol level, systolic blood pressure, and waist circumference. [10]

In the current study we examined the presence of insulin resistance and most of the metabolic and clinical characteristics associated with this syndrome. The absence of studies in our country as in other poor Latin American countries, make interesting to design the current study about this emerging problem in comparison with another countries.

The objective of our study was to determine the association between insulin-resistance and components of the metabolic syndrome in a group of Bolivian obese children and adolescents. The second objective was determining the relation of acanthosis nigricans and insulin-resistance.

## Methods

### Study population

We studied obese children and adolescents beginning in August 2006 to August 2007 in the Pediatric Center Albina Patiño, reference hospital of the city of Cochabamba, Bolivia. Subjects were eligible if they were healthy, were between 4 and 18 years of age, and had a body-mass index that exceeded the 95th percentile for their age and sex (according to CDC norms).

Exclusion criteria were the presence of diabetes and the use of drugs that alters blood pressure or glucose or lipid metabolism. Parents form consent was obtained before the initial diagnostic tests were performed.

The study protocol was previously reviewed and approved by the ethics and research committee of the Centro Pediatrico Albina Patiño.

### Procedures

Height and weight were measured in the first visit of patient, calculating the body mass index (BMI) according to the formula (weight in kilograms divided by the square of the height in meters). Additionally waist and hip measurements were obtained in order to calculate the waist-hip ratio (WHR).

Blood pressure was also controlled in the initial and the final part of the medical visit, calculating a mean between the two measurements as the final result.

A meticulous physical examination was performed looking for characteristic abnormalities in patients with metabolic syndrome, specifically acanthosis nigricans in the back of the neck and axillary area.

Baseline blood samples were obtained from subjects while they were fasting in order to measure levels of glucose, insulin and lipids. All of the patients were previously indicated for fasting at least 10 hours before the blood sample was obtained. An oral glucose-tolerance test was then performed with the administration of 1.75 g of glucose per kilogram of body weight (maximal dose, 75 g) followed by measure of insulin and glucose two hours after glucose ingestion.

### Biochemical analysis and definitions

Plasma glucose levels were measured with the enzymatic colorimetric method GOD-PAP. In the other side insulin was calculated and measured by a radioimmunoanalysis method. We considered hyperinsulinism as the presence of basal insulin > 143.5 pmol/liter and > 430.5 pmol/liter after the glucose tolerance test according to WHO criteria, or insulin > 86.1 pmol/liter with glucose < 3.3 mmol/liter. [11,12]

The data for insulin resistance were based on the homeostatic model assessment (HOMA), calculated as the product of the fasting plasma insulin level (in microunits per milliliter) and the fasting plasma glucose level (in millimoles per liter), divided by 22.5. Scores ordinarily range from 0.3 to 10, taking scores > 3.5 as the existence of insulin resistance and scores < 3.5 as insuline sensitive.

Cholesterol, triglycerides, high density lipoprotein (HDL) and low density lipoprotein (LDL) was also calculated in all patients. Abnormalities in the fasting levels of triglycerides and high-density lipoprotein (HDL) cholesterol were adjusted for age and sex (> 95th percentile for triglycerides; < 5th percentile for HDL cholesterol). Impaired glucose tolerance was defined as a glucose level greater than 7.8 mmol per liter but less than 11.1 mmol per liter at two hours.

Metabolic syndrome was considered if three or more of the following criteria were present: BMI > 95th percentile, triglyceride level above the 95th percentile, HDL cholesterol level below the 5th percentile, systolic or diastolic blood pressure above the 95th percentile, and impaired glucose tolerance. [20-24]

### Statistical analysis

All data were introduced in the JMP 6.0 statistical discovery program for analysis of means, medians an standard deviations. Fisher's exact test was used to determine statistical differences in presence of metabolic syndrome criteria according to sex and to correlate the association among insulin resistance presence or absence and metabolic syndrome criteria plus acanthosis nigricans.

T test was calculated to determine statistical significance (p < 0.05) in clinical and biochemical characteristics according to sex.

## Results

A total of 13668 pediatric visits were performed between august 2006 to August 2007 finding 155 patients with the diagnosis of obesity. Only 61 patients of the 155 concluded with all the biochemical analysis required for the final analysis. No significant differences were found between children that concluded the study and those who did not.

All the clinical and demographic characteristics are shown in table 1 where the sex prevalence is almost identical with 31(50.8%) corresponding to females and 30(49.2%) to males.

**Table 1:** Clinical characteristics according to sex.

**Characteristic**	**Females (n = 31)**	**Males (n = 30)**	**P value ¶**
	(Mean ± SD)	(Mean ± SD)	
Age (years)	8.9 ± 2.6	9.7 ± 3.1	0.1656
Weight (Kg)	50.7 ± 14.4	50.9 ± 18.5	0.4889
Height (m)	1.35 ± 0.15	1.39 ± 0.15	0.1289
Body-mass index	26.7 ± 3.7	25.5 ± 3.1	0.9151
Systolic pressure (mmHg)	104.1 ± 12.5	103.1 ± 13.2	0.6140
Waist-hip ratio	0.92 ± 0.05	0.95 ± 0.06	0.0468

¶ T test

Racial variables were not considered because all of the patients correspond to Hispanic race.

Biochemical characteristics including HDL, LDL, triglycerides, glucose and insulin levels are summarized according to sex in table 2 where a substantial difference between means were found as expected. No significant statistical significance was found between sex except for HDL levels (p = 0.0360).

**Table 2:** Biochemical characteristics according to sex.

**Characteristic**	**Females (n = 31)**	**Males (n = 30)**	P value *
	(Mean ± SD)	(Mean ± SD)	
Fasting insulin(pmol/liter)	98.2 ± 75.3	144.9 ± 125.5	0.0438
Fasting Glucose (mmol/liter)	4.1 ± 0.85	4.25 ± 0.56	0.2353
Insulin resistance (HOMA)	2.6 ± 2.2	3.3 ± 1.81	0.1013
Total cholesterol (mmol/liter)	4.31 ± 1.07	4.64 ± 1.22	0.1332
Triglycerides (mmol/liter)	1.20 ± 0.43	1.42 ± 0.55	0.0518
HDL (mmol/liter)	0.88 ± 0.21	0.96 ± 0.24	0.0360
LDL (mmol/liter)	3.06 ± 0.63	3.03 ± 0.59	0.5810

* T test

Metabolic profile and metabolic syndrome criteria according to sex are described in table 3, where the presence of insulin resistance is higher in males (50%) than females (29%), but without statistical significance (p = 0.1). Metabolic syndrome criteria in obese patients were observed in the next descendent order, HDL < 5th percentile (55.7%), triglycerides > 95th percentile (42.6%), systolic blood pressure > 95th percentile (24.5%) and impaired glucose tolerance (8.2%). A metabolic syndrome was diagnosed in 22(36.07%) of the total number of patients corresponding most of them to male group.

**Table 3:** Metabolic profile and metabolic syndrome criteria according to sex.

**Characteristic**	**Females (n = 31)**	**Males (n = 30)**	**Total**	**P Value †**
	n (%)	n (%)	n (%)	
Impairment of glucose tolerance	2 (6.45)	3 (10)	5 (8.2)	0.6713
BMI > 95th percentile	31 (100)	30 (100)	61 (100)	-
Systolic blood Pressure > 95th Percentile	10 (32.2)	5 (16.6)	15(24.59)	0.2351
HDL < 5th percentile	22 (70.9)	12(40)	34 (55.7)	0.0209
Triglycerides > 95th percentile	11 (35.48)	15 (50)	26 (42.6)	0.3056
Insulin resistance (HOMA)	9 (29)	15 (50)	24 (39.4)	0.1196
Metabolic syndrome	10 (32.2)	12 (40)	22 (36.07)	0.5996
Achantosis nigricans Presence	17 (27.8)	20 (32.7)	37 (60.6)	0.4345

† Fisher's Exact Test (two-tailed test)

In table 4, we correlate the metabolic syndrome criteria findings with the presence of resistance to insulin. The correlation between insulin resistance and high blood pressure was statistically significant (p = 0.0148) as with high triglyceride levels (p = 0.002).

**Table 4:** Prevalence of components of metabolic syndrome according to insulin-resistance

**Criteria**	**Insulin resistance (n = 24) **	**Insulin sensitive (n = 37)**	**P value‡**
	N° (%)	N° (%)	
Glucose impairment tolerance	2 (8.3)	3 (8.1)	0.66
High systolic blood Pressure	10 (41.6)	5 (13.5)	0.0148
Low HDL levels	13 (54.1)	21 (56.7)	0.67
High Triglycerides	16 (66.6)	10 (27)	0.0025
Achantosis nigricans	17 (70.8)	20 (54)	0.14

‡ Fisher's Exact Test. (two-tailed test)

Acanthosis nigricans was found in (70%) of patients resistant to insulin, but it was also present in 54% of patients sensitive to insulin (p = 0.14).

## Discussion

The lack of previous studies about metabolic syndrome in Bolivia makes it interesting to know the actual situation in one of the poorest country in South America. The number of patients was not the ideal to try to show the prevalence in the entire country, but it would be an initial point for future studies.

The economical situation was an important determinant factor in our study since many obese children were not included because of lack of enough money for medical visits and some of the diagnostic test that were not financed by research committee of the hospital.

Insulin resistance was found in 39.4% of obese children and adolescents. This rate is similar as other found in studies conducted in developing countries like France, Italy, Spain and United States of America. [15-19]

There are no clear definition for metabolic syndrome in children and adolescents, existing different criteria used according to the place or population of the study. [20-24] NCEP-ATP III definition with modified cut-off values for youths was considered for the diagnosis of metabolic syndrome in the current study.

Waist-hip ratio was also determined; however, it is important to know that neither insulin concentration nor centrally obesity is considered like criteria in children groups in contrast with adolescents groups where some definitions include WHR as criteria. [22,25,26] This association was demonstrated specially in studies conducted in adult patients with metabolic syndrome. [13,14] In the current study WHR was the only one characteristic that demonstrated statistical significance according to sex (p = 0.046).

The metabolic syndrome was found in 36% of studied patients, with a slightly predilection in males but without statistical significance (p = 0.59). Lipidic abnormalities account for most of the criteria of definition metabolic syndrome. High blood pressure and impairment of glucose tolerance frequencies are still low in the current investigation as expected in comparison with another studies.

In pigmented races, acanthosis nigricans (AN) is an important early manifestation of the obesity syndrome. AN helps identify persons at particular risk of developing the obesity syndrome, dyslipidaemia, hypertension and insulin resistance. Recognition of AN, therefore, offers important opportunities for health screening and preventative medicine. [27-31]

Acanthosis nigricans was found in 70% of the insulin resistant group of patients, but it was also present in the 54% of sensitive insulin group. This factor was important in the lack of statistical significance of this association. The existent relation between obesity and AN is more prevalent in this study as in other published investigations. [32-34] A stronger correlation was found between high blood pressure and high triglyceride levels in patients with insulin resistance. This association was also determined in some studies. [35,36]

It is clear that the incidence and prevalence of metabolic syndrome and obesity tend to increase with the passage of time, becoming an alarming problem of public health. Therefore, all health politics and efforts should be conducted for nutritional education, sports practice, change dietary habits (increase the amount of fiber, reduce the intake of junk food and saturated fat) that may have important role in the reduction of obesity and metabolic syndrome prevalence.

## Conclusion

Our sample has a prevalence of 36% among children and adolescent with obesity in Bolivia. Insulin resistance is very common among children and adolescents with obesity, significantly associated with high blood pressure and high triglycerides levels. Obesity as malnourishment is an alarming problem of public health even in developing countries.

## Abbreviations

National Cholesterol Education Program-Adult Treatment Panel: NCEP-ATP III; High density lipoprotein: HDL; Low density lipoprotein: LDL; Very low density lipoprotein: VLDL; waist-hip ratio: WHR; homeostatic model assessment: HOMA; body mass index: BMI; acanthosis nigricans: AN.

## Competing interests

The authors declare that they have no competing interests.

## Authors' contributions

MC designed the study and collected the data. CGT analyzed the data, participated in data interpretation and wrote most of the article in English language. SR, MM participated in the design of the study. All authors critically reviewed the manuscript.

## Pre-publication history

The pre-publication history for this paper can be accessed here:


